# Application of YOLO11 Model with Spatial Pyramid Dilation Convolution (SPD-Conv) and Effective Squeeze-Excitation (EffectiveSE) Fusion in Rail Track Defect Detection

**DOI:** 10.3390/s25082371

**Published:** 2025-04-09

**Authors:** Weigang Zhu, Xingjiang Han, Kehua Zhang, Siyi Lin, Jian Jin

**Affiliations:** 1 College of Engineering, Zhejiang Normal University, Yingbin Avenue, Jinhua 321005, China; zwg1654@zjnu.edu.cn (W.Z.); hxj90626@zjnu.edu.cn (X.H.); jjbbetter@zjnu.edu.cn (J.J.); 2 Key Laboratory of Urban Rail Transit Intelligent Operation and Maintenance Technology & Equipment of Zhejiang Province, Zhejiang Normal University, Yingbin Avenue, Jinhua 321005, China; 3 School of Computer Science and Technology, Zhejiang Normal University, Yingbin Avenue, Jinhua 321005, China; lsysxka041@zjnu.edu.cn

**Keywords:** rail track defect detection, deep learning, YOLO11, SPD-Conv, EffectiveSE

## Abstract

With the development of the railway industry and the progression of deep learning technology, object detection algorithms have been gradually applied to track defect detection. To address the issues of low detection efficiency and inadequate accuracy, we developed an improved orbital defect detection algorithm utilizing the YOLO11 model. First, the conventional convolutional layers in the YOLO (You Only Look Once) 11backbone network were substituted with the SPD-Conv (Spatial Pyramid Dilation Convolution) module to enhance the model’s detection performance on low-resolution images and small objects. Secondly, the EffectiveSE (Effective Squeeze-Excitation) attention mechanism was integrated into the backbone network to enhance the model’s utilization of feature information across various layers, thereby improving its feature representation capability. Finally, a small target detection head was added to the neck network to capture targets of different scales. These improvements help the model identify targets in more difficult tasks and ensure that the neural network allocates more attention to each target instance, thus improving the model’s performance and accuracy. In order to verify the effectiveness of this model in track defect detection tasks, we created a track fastener dataset and a track surface dataset and conducted experiments. The mean Average Precision (mAP@0.5) of the improved algorithm on track fastener dataset and track surface dataset reached 95.9% and 89.5%, respectively, which not only surpasses the original YOLO11 model but also outperforms other widely used object detection algorithms. Our method effectively improves the efficiency and accuracy of track defect detection.

## 1. Introduction

As an important means of transportation, trains have shown remarkable development in recent years. This progress has not only promoted economic exchanges, but also promoted the booming tourism industry, which has significantly improved people’s quality of life. Track defects have a direct and serious impact on train safety. For example, track surface defects may lead to poor wheel–rail contact, and track fastenings defects may lead to unstable rail fixation, affecting the stability of trains and increasing the risk of derailment [1].

At present, there are a variety of methods used to track defect detection, ranging from traditional manual detection to advanced automated detection. Manual inspection relies on the experience and skills of the inspectors; inspection efficiency is low and cannot cover a large area of the track. In addition, some researchers use instruments such as ultrasonic, magnetic particles, eddy currents, and other detection equipment; however, this leads to problems such as equipment professionalism and the large number of prior data that need to be processed, and the investment cost is high. With the development of automated inspection, people have begun to use unmanned vehicles or drones, combined with vision sensor and image recognition technology, to detect track defects.

### 1.1. Track Detection Based on Deep Learning

Deep learning is an excellent artificial intelligence method, and the field of orbital defect detection has begun to explore some methods based on deep learning. Gibert et al. [2] proposed a deep multi-task learning method for defect detection with few defect images, and applied it to defect detection in track sleepers and fasteners, improving the accuracy of detection. Acikgoz et al. [3] proposed a multi-scale residual convolutional network (MSR-convnet) model used to classify different types of rail track defects. Lu et al. [4] proposed a SCueU-Net model, combined with a U-Net [5] segmentation network and defect location method, which was applied to the task of high-speed rail defect detection, and the detection accuracy of this method was 99.76%. Chandran et al. [6] proposed an image processing algorithm that can accurately locate fasteners while removing redundant information in fasteners. Convolutional neural networks (CNN) and ResNet-50 algorithms are used for classification purposes. The accuracy of these two algorithms in the training and verification stage is more than 98%. Kim et al. [7] developed a segmentation model using an improved full convolutional neural network to automatically detect and segment defects on the orbital surface. Aydin et al. [8] processed the enhanced image with local binary method, determined the position of the retainer, and then used a support vector machine as the improved lightweight convolutional neural network (LCNN) that classifies the defects; the detection accuracy reached 99.7%. Jin et al. [9] used Markov random fields and a faster region proposal convolutional neural network (Faster RCNN) to construct a deep multiple model. The fast and accurate edge segmentation of rail surface defects was achieved. This method has good robustness and can resist various noise and lighting conditions. In 2020, Ye et al. [10] designed a differential feature fusion convolutional neural network (DFF-Net) to detect objects in rail traffic to stop rail accidents, which outperformed the latest state-of-the-art detectors with an mAP of 90.12%. In 2022, Kapoor et al. [11] used 2DSSA (2D Singular Spectrum Analysis) in the design of a network of deep classifiers that can efficiently identify objects on railway tracks in front of trains.

### 1.2. Track Detection Based on YOLO

The You Only Look Once (YOLO) [12] model series, a deep learning-based object detection framework, has been widely used in railway track defect detection. Qi et al. [13] introduced a novel network model designated MYOLOv3-Tiny. This model is characterized by an efficiently designed backbone network, which enhances the extraction of features related to track fasteners while simultaneously decreasing computational complexity. As a result, it achieves improved detection accuracy and enhanced real-time performance. Hsieh et al. [14] built offline and online track fastening detection systems based on YOLOv3 and YOLOv4-tiny in 2020 and 2022, respectively. Wang et al. [15] established a railway fastener defect detection method that integrates the YOLOv5 model with the FPGM (Filter Pruning via Geometric Median) algorithm, applying it to the process of model pruning. Feng et al. [16] enhanced YOLO through the incorporation of a feature pyramid, resulting in high-performance detection outcomes. Zhao et al. [17] introduced the RDD-YOLO model specifically for the detection of defects on steel surfaces. By optimizing the backbone network, implementing a double-feature pyramid network, and utilizing a decoupled head structure, this model markedly improves detection accuracy and enables the precise localization of defects on steel surfaces. Hu et al. [18] proposed a YoloX-Nano model with a coordinate attention mechanism, which can detect fastener defects more quickly. In 2022, Wang et al. [19] proposed YOLOv5s VF, a revolutionary fault detection network for orbital surfaces. The V-CBAM includes two modules, SSA (sharpened spatial attention) and FCAM (adaptive channel attention), and the results show that the overall speed of YOLOv5s VF is 114.9 fps (frames per second) and the accuracy is 93.5%, which exceeds the current object recognition methods on the orbital surface. Li et al. [20] not only added the attention mechanism and feature pyramid to the YOLOv5 algorithm, but also used the K-means algorithm to recluster the data set, improve the anchor frame’s ability to locate defects, and attain improved detection accuracy and increased detection speed.

In general, YOLO algorithm has a high real-time performance, is suitable for processing large-scale data, and has good target location and classification capabilities, but the detection effect of small-scale targets and dense targets is poor and its accuracy may not be high compared with some complex scenes.

The YOLO11 model, representing the latest iteration in the YOLO series released by Ultralytics, incorporates an enhanced backend and neck architecture. This significantly improves its feature extraction capabilities, thereby facilitating more accurate object detection and the execution of complex tasks. Additionally, it introduces a refined architectural design along with an optimized training process, which facilitates quicker processing speeds while preserving an optimal equilibrium between accuracy and performance.

In this study, we employed YOLO11 to detect rail fasteners and surface defects, and optimized the YOLO11 algorithm in accordance with the characteristics of the rail dataset. We substituted the original convolutional layers in the YOLO11 backbone with SPD (Spatial Pyramid Dilation)-Conv building blocks to improve the model’s sensitivity to medium and small targets. Additionally, we introduced the EffectiveSE attention mechanism, integrating this module into the backbone network to enable the model to leverage information from features at different layers, thereby improving its feature representation capabilities. Finally, we added a small target detection head in the neck network to bolster detection performance across various target scales, allowing for the more accurate identification of rail defect features in complex scenarios.

Through careful data set annotation and rigorous validation process, our algorithm achieved the accurate location and identification of track defects. These enhancements significantly improved the overall detection performance of the algorithm.

## 2. Improved Algorithm Design

The architecture of YOLO11 is designed to optimize speed and accuracy, and builds upon improvements introduced in earlier versions of YOLO, such as YOLOv8, YOLOv9, and YOLOv10. YOLO11’s major architectural innovations revolve around its use of C3K2 (a faster CSP module) blocks, SPFF (Spatial Pyramid Pooling–Fast) modules, and a C2PSA (Channel-wise Positional Self Attention) block, improvements that enhance its ability to process spatial information while maintaining high-speed reasoning. The YOLO11 architecture series covers five different scale models, namely YOLO11n, YOLO11s, YOLO11m, YOLO11l, and YOLOx. Its network depth and width increase in turn, providing a variety of options regarding the model complexity and computing resource requirements.

The YOLO11n model features the most streamlined design in terms of network depth and width, which not only yields the smallest model size but also enhances its real-time processing capability to the highest level. Given the stringent requirements for real-time performance in rail defect detection tasks, we selected YOLO11n as the model for this study.

YOLO11 comprises three primary components: the backbone network, the neck network, and the head network. The backbone network is tasked with feature extraction from input images of the size 640 × 640 × 3. This is the basis of the entire model and determines the amount of image information that can be captured by the model, which mainly includes four modules: Conv, C3k2, SPPF, and C2PSA. These modules are used to gradually reduce the spatial resolution of the image and increase the number of channels, thus generating feature-rich maps.

The neck network is positioned between the backbone network and the head network, utilizing a PAN (Path Aggregation Network) structure to facilitate the additional processing of the feature map derived from the backbone network. The main purpose of this section is to enhance feature representation and provide better input to the head network.

**Figure 1:**
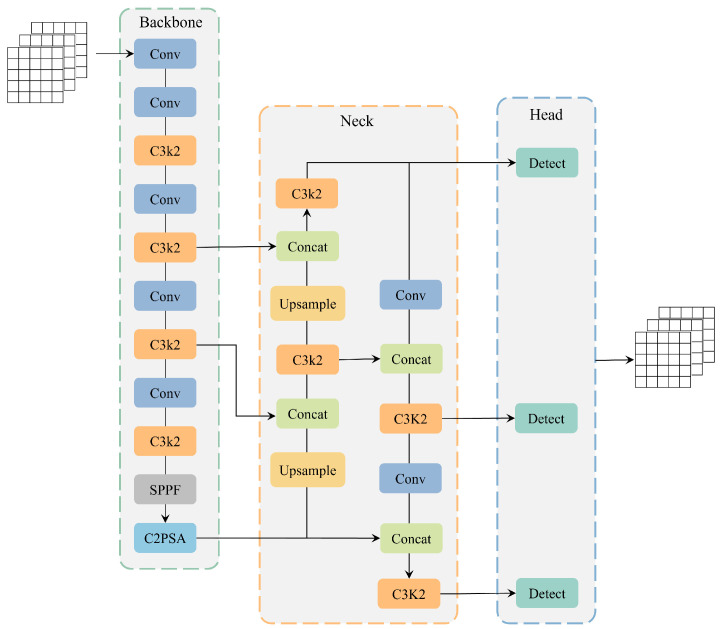
The original architecture of YOLO11.

The head network is responsible for the final target detection prediction according to the feature map provided by the neck network. It consists of three independent detection heads, which are used to process feature maps of large, medium, and small sizes to meet the detection requirements of different targets. As a result, the network outputs a high-dimensional tensor containing class, confidence, and position coordinate information. Figure 1 shows the network structure of YOLO11.

**Figure 2:**
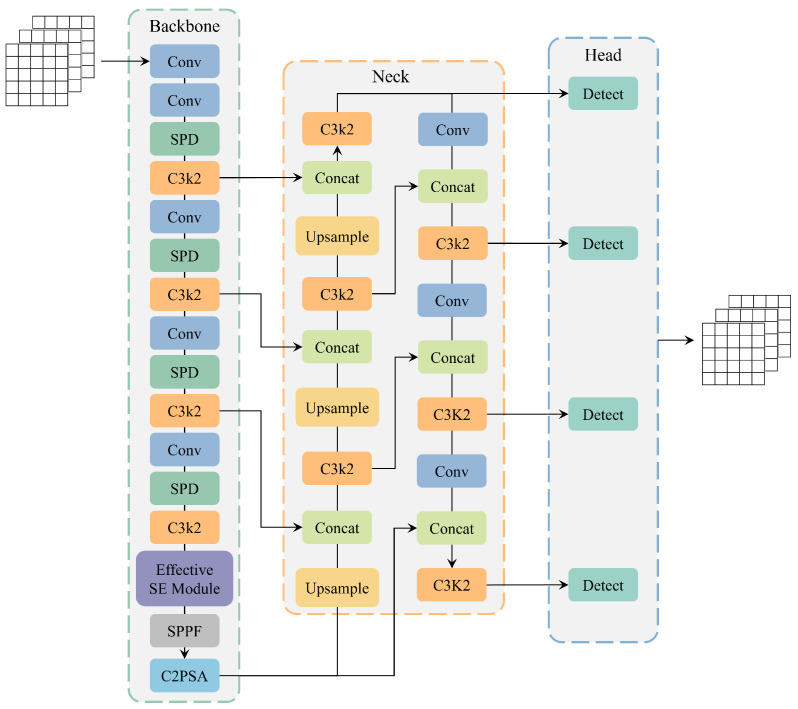
The improved architecture of YOLO11.

However, the original YOLO11n model has some problems in the detection of track defect data set, such as false detection, missed detection, and insufficient detection accuracy. Based on the original YOLO11 model, this paper introduces the SPD-Conv building block, EffectiveSE attention mechanism, and improvements in small target detection. These enhancements significantly improve the algorithm’s ability to recognize small targets and low-resolution images from the perspectives of feature extraction, channel attention mechanism, and object detection, resulting in a new rail defect detection model that achieves the optimal overall detection performance. This work not only solves the defects in the existing model, but also significantly enhances the model’s detection accuracy, and further progresses the field of track defect detection. The improved YOLO11 model is shown in Figure 2.

### 2.1. SPD-Conv Building Block

When dealing with low-resolution images and small targets, the original YOLO11 model may face certain limitations, resulting in a decrease in its detection performance. Since orbit defects often involve a large number of small defects, the original YOLO11 model hasa poor detection effect on such targets. SPD-Conv enhances the model’s ability to handle small objects and low-resolution images, and some researchers have used it to improve previous YOLO models. In order to address these issues, we pioneered the integration of the SPD-Conv module after each convolution layer of the YOLO11 model, enhancing its performance. By removing step convolution and pooling operations, we ensure that the learnable information is retained throughout the downsampling process. Thus, the model’s performance across various visual tasks is enhanced.

**Figure 3:**
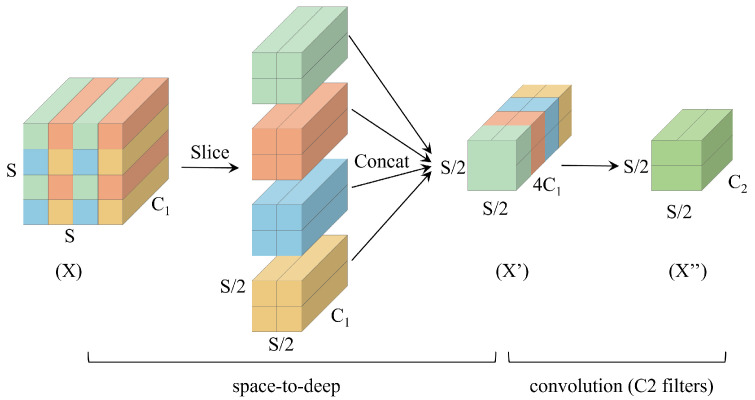
The architecture of SPD-Conv.

SPD-Conv [21] is composed of an SPD layer in conjunction with a non-strided convolution layer. SPD performs a sparse point sampling on the feature map in CNN and the entire CNN architecture. As shown in the Figure 3, for any intermediate feature graph X of size S × S × C1, a set of sub-feature graphs is generated, as shown in Equation (1). Taking scale = 2 as an example, feature graph X is subsampled and divided into four subgraphs $G_{0,0}$, $G_{1,0}$, $G_{0,1}$, $G_{1,1}$, each of which has a size of $\left(S/2,S/2,C_{1}\right)$. The four subgraphs are joined together in the channel direction to form a new feature graph X’ of size $\left(S/2,S/2,4C_{1}\right)$. The dimensions of the X’ feature map are diminished to one half of the original length and width, while the number of channels is increased fourfold. This downsampling method is beneficial for low-resolution images.

After the transformation through the SPD layer, a non-strided (stride = 1) convolutional layer with C2 filters (where $C_{2}<2^{2}C_{1}$) is applied. This configuration is intended to preserve all discriminative feature information, ultimately resulting in a feature map X′′ of size $\left(S/2,S/2,C_{2}\right)$.

(1) $$\begin{array}{c} G_{0,0}=X\left[0:S:scale,0:S:scale\right]\\ G_{1,0}=X\left[1:S:scale,0:S:scale\right]\\ G_{0,1}=X\left[0:S:scale,1:S:scale\right]\\ G_{1,1}=X\left[1:S:scale,1:S:scale\right]\\ X^{\prime }=G_{0,0}\left|\right|G_{1,0}\left|\right|G_{0,1}\left|\right|G_{1,1} \end{array}$$

### 2.2. EffectiveSE Attention Mechanism

The EffectiveSE [22] module is an enhanced channel attention mechanism designed to maintain model performance while reducing computational complexity and mitigating issues such as information loss. By adaptively adjusting the weights of each channel, EffectiveSE significantly improves the network’s ability to recognize various targets in complex scenes, thereby enhancing detection accuracy and robustness.

The EffectiveSE architecture consists of two primary components: Squeeze and Excitation. The Squeeze component employs a Global Average Pooling layer to condense the feature map of each channel into a scalar value, thereby effectively capturing global spatial information. The Excitation component employs a Multi-Layer Perceptron (MLP) to learn the weights for each channel. Initially, the scalar values produced by the Squeeze component are processed through a fully connected layer to generate channel weights. These weights are then multiplied by the original feature maps to produce weighted feature maps, enhancing the expressiveness of important features. The operations of EffectiveSE are represented by Equations (2)–(4).

In Equations (2)–(4), *W* and *H* represent the width and height of the input feature map, respectively, while c denotes the number of channels in the feature map $X_{ijc}$. First, for the input feature map, global average pooling is performed using Equation (2) to obtain the weights for each channel. Subsequently, these weights $W_{c}$ are mapped to a new activation value $f^{\prime }\left(W_{c};\theta \right)$ through a fully connected layer, followed by a sigmoid activation, as illustrated in Equation (3). Finally, the weights $S_{c}$ for each channel are multiplied by the original feature map $X_{ijc}$ to generate the weighted feature map $Y_{ijc}$, as shown in Equation (4).

**Figure 4:**
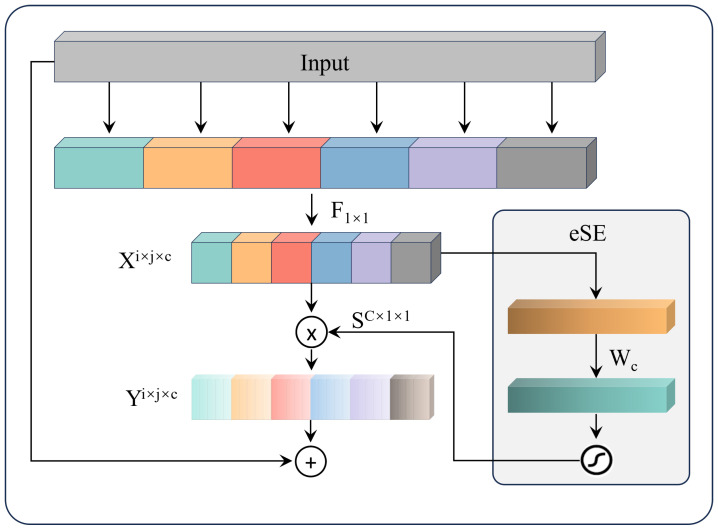
The architecture of EffectiveSE.

The EffectiveSE attention mechanism utilizes only a single fully connected layer, preserving the channel dimension and avoiding information loss. This simplified structure not only reduces computational overhead but also enhances the overall efficiency of the model through more effective computations. A structural diagram of the EffectiveSE module is presented in Figure 4.

(2) $$W_{c}=\frac{1}{H\times W}\sum_{i=1}^{H}\sum_{j=1}^{W}X_{ijc}$$

(3) $$S_{c}=\sigma \left(f^{\prime }\left(W_{c};\theta \right)\right)$$

(4) $$Y_{ijc}=S_{c}X_{ijc}$$

### 2.3. Small Object Detection Head

The original YOLO11 network features three detection heads of different sizes. The P3 detection head primarily focuses on detecting larger objects, while the P4 detection head is responsible for medium-sized targets and the P5 detection head is explicitly tailored to the detection of small objects. This design enables YOLO11 to be more flexible and efficient when handling targets of varying sizes. Nevertheless, in practical applications, the pixel ratio of the target defects tends to be relatively low. The detection heads provided by YOLO11 may struggle with these small targets; this results in challenges such as missed detections and suboptimal detection performance.

To address this challenge, this study introduces a new P2 small object detection head aimed at enhancing the model’s detection capabilities. The P2 detection head is specifically optimized for smaller targets, enabling it to effectively recognize objects that occupy a minimal number of pixels within the image.

**Figure 5:**
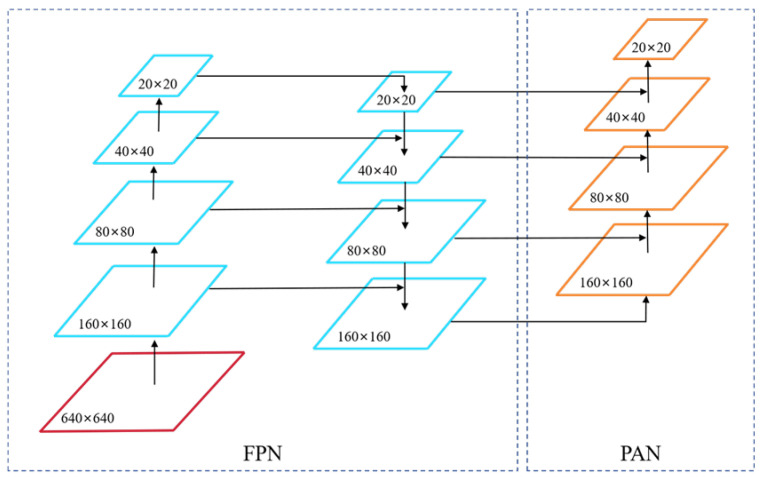
The architecture of the small object detection head.

When the input image size is 640 × 640, the three detection heads included in the YOLO11 output detection feature maps gave sizes corresponding to P3, P4, and P5, which are 80 × 80, 40 × 40, and 20 × 20, respectively. These heads are designed to detect targets larger than 8 × 8 pixels, 16 × 16 pixels, and 32 × 32 pixels, respectively. The additional P2 detection head processes and outputs a feature map of size 160 × 160, enabling the model to recognize smaller-sized targets. This high-resolution feature map significantly enhances the small object detection capabilities, particularly in complex tracking scenarios, allowing for the effective capture of defects in fasteners and surfaces. The P2 small object detection head is integrated with the three detection heads provided by YOLO11, forming a multi-scale detection architecture, as illustrated in Figure 5.

## 3. Experiment and Results

### 3.1. Data Collection and Preparation

**Figure 6:**
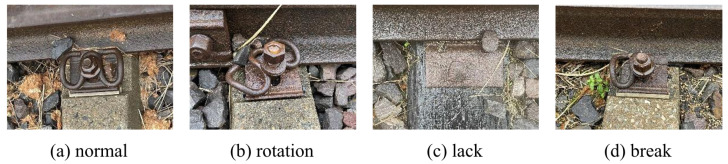
Example images of track fastener dataset.

**Figure 7:**
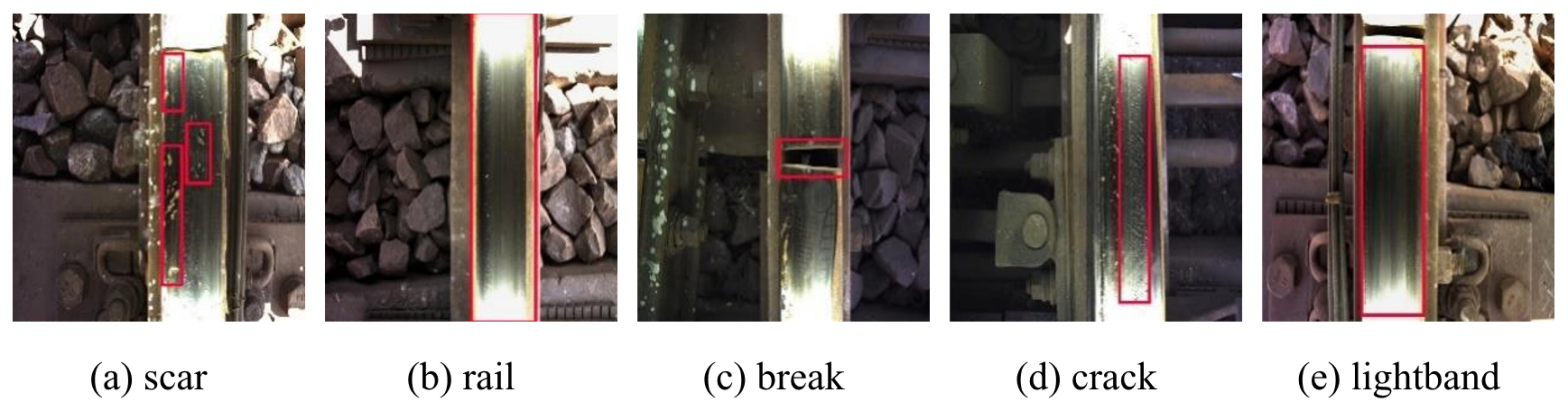
Example images of track surface dataset. (The red box marks the specific location of the target. There is no red box in the original image).

Railway inspection primarily focuses on surface defect identification and fastener detection [23,24]. The dataset utilized in this study comprises a track fastener dataset and a track surface dataset. Currently, datasets related to track defects are relatively scarce, and due to confidentiality principles, most railway data are not publicly accessible. Consequently, part of our data was sourced from publicly available datasets online, while another part consisted of real track images captured by a color vision sensor with a resolution of 1920 × 1080. This sensor was mounted on a rail vehicle and collected 1959 raw images from various sections of the railway site, saving them in JPG format. To increase the number of images while ensuring that the sample size ratios of each category in the dataset closely reflect real-world scenarios, we performed various data augmentation operations. These operations included image position transformations, noise addition, and color jittering. Additionally, we simulated various lighting conditions and occlusion scenarios, which account for approximately one-third of the total samples. The dataset also includes common railway field interferences, such as rainy conditions and low-light tunnel scenarios. Subsequent experiments demonstrate that differences in the sample sizes of the dataset do not affect the model’s detection performance. After screening and labeling, these images were divided into training, validation, and test sets in an 8:1:1 ratio. The track fastener dataset includes four detection categories—normal, rotation, lack, and break—whereas the track surface dataset contains five detection categories—scars, cracks, breaks, lightbands, and rails. The total number of images and instances for each category is detailed in Table 1 and Table 2. Figure 6 and Figure 7 present example images from both datasets.

### 3.2. Experimental Equipment

In this study, all experiments were conducted on a high-performance workstation, the hardware configuration and software environment of which are detailed in Table 3. To ensure the originality of the results, no pre-trained weights were employed during the model training process. All input images were consistently resized to a specification of 640 × 640 × 3. The batch size utilized during training was set to 32, and the entire training process lasted for 300 epochs. All other training parameters were maintained at their default settings.

### 3.3. Performance Metrics

When evaluating the object detection algorithms, we primarily focused on two aspects: detection accuracy and model complexity. Detection accuracy is measured by metrics such as Precision, Recall, and Average Precision (AP), which assess the model’s recognition capability. Model complexity, on the other hand, is evaluated through the size of the model’s weight files and the total number of parameters, reflecting its resource consumption. Together, these factors determine the model’s performance and feasibility in practical applications.

Precision is defined as the ratio of correctly classified target boxes to the total number of detected boxes, serving as a crucial indicator of the accuracy of the object detection algorithm. Recall represents the proportion of correctly detected boxes among all actual target boxes, measuring the comprehensiveness and retrieval capability of the algorithm.

Average Precision (AP) is a holistic evaluation metric employed to objectively evaluate the performance of object detection models across different Intersection over Union (IoU) thresholds. AP is derived by calculating the area under the Precision–Recall curve. Specifically, for each object category, a set of Precision and Recall values is computed based on the different IoU thresholds. This set of values is then interpolated to calculate the area under the curve, representing the AP for that category.

Mean Average Precision (mAP) serves as a comprehensive evaluation metric obtained by averaging the areas under the Precision–Recall curves for different categories. mAP is one of the most widely used performance evaluation metrics in the field of object detection, providing an overall assessment of the model’s accuracy and recall. Typically, mAP is computed across a series of IoU thresholds, such as 0.5, 0.75, and 0.95, to consider performance across varying thresholds.

Let us assume that the number of true positive samples in the prediction results is denoted as TP (True Positives), the number of false positives is represented as FP (False Positives), and the number of false negatives is indicated as FN (False Negatives), with i denoting the category in the dataset. Based on these definitions, the equations for calculating Precision (P), Recall (R), and Mean Average Precision (mAP) are as follows:

(5) $$\mathrm{Precision}=\frac{TP}{TP+FP}$$

(6) $$\mathrm{Recall}=\frac{TP}{TP+FN}$$

(7) $$\mathrm{AP}=\int_{0}^{1}P\left(R\right)\;dR$$

(8) $$\mathrm{mAP}=\frac{1}{C}\sum_{i=1}^{C}AP_{i}$$

### 3.4. Experimental Results and Analysis

We tested the improved model on the rail fastening dataset and the rail surface dataset, with the mAP@0.5 metrics for each category shown in Table 4 and Table 5. Combining the insights from Table 1 and Table 2, it is evident that the sample sizes across categories do not correlate with detection performance. For instance, the track fastening dataset has the highest number of normal samples, yet its detection accuracy is similar to that of other samples. In the track surface dataset, the number of scar samples far exceeds that of break samples; however, due to the small size of the scar samples, the detection accuracy is quite low. Despite the differences in sample sizes, the model effectively addresses class imbalance and maintains a high detection accuracy.

#### 3.4.1. Ablation Experiment

To systematically assess the performance gains brought about by the introduction of the SPD-Conv block, the EffectiveSE attention mechanism, and the small object detection head in the YOLO11n model, this study meticulously designed and executed a series of ablation experiments. These experiments were carried out based on the track fastener dataset and the track surface dataset, using the original YOLO11n model as a baseline for comparison. We comprehensively evaluated the impact of each improvement on model performance by examining metrics such as model size, parameter count, Precision (P), Recall (R), mAP@0.5, and mAP@0.5:0.95.

The experiments employed various module combination strategies to explore the synergetic effects among the different components. The results indicate that each component, whether used individually or in combination, enhances model performance from different dimensions. For example, the SPD-Conv module preserves more spatial details by avoiding information loss in traditional downsampling operations; the EffectiveSE module enhances the channel attention mechanism, enabling the model to better focus on key features. The combination of these two modules allows the model to retain rich spatial information while efficiently leveraging the channel attention mechanism, thereby significantly improving detection performance. However, the optimal overall performance is achieved when all three modules work together. Specifically, on the track fastener dataset, the improved model demonstrated a 2.7% increase in Precision (P), a 0.9% increase in Recall (R), a 1.8% increase in mAP@0.5, and a significant 5.8% improvement in mAP@0.5:0.95 compared to the baseline model. Similarly, on the track surface dataset, P, R, mAP@0.5, and mAP@0.5:0.95 improved by 1.4%, 0.8%, 0.9%, and 1.7%, respectively.

To validate the model’s generalization ability, we conducted experiments on the test sets of the track fastener dataset and the track surface dataset, which were not involved in the model’s training. The original YOLO11 achieved mAP@0.5 scores of 94.0% and 87.3% on the two test sets, while the improved algorithm achieved mAP@0.5 scores of 95.4% and 88.6%, respectively. These results indicate that the model performs excellently on new data outside of the training set, demonstrating that the method effectively enhances the model’s generalization ability in new scenarios.

**Figure 8:**
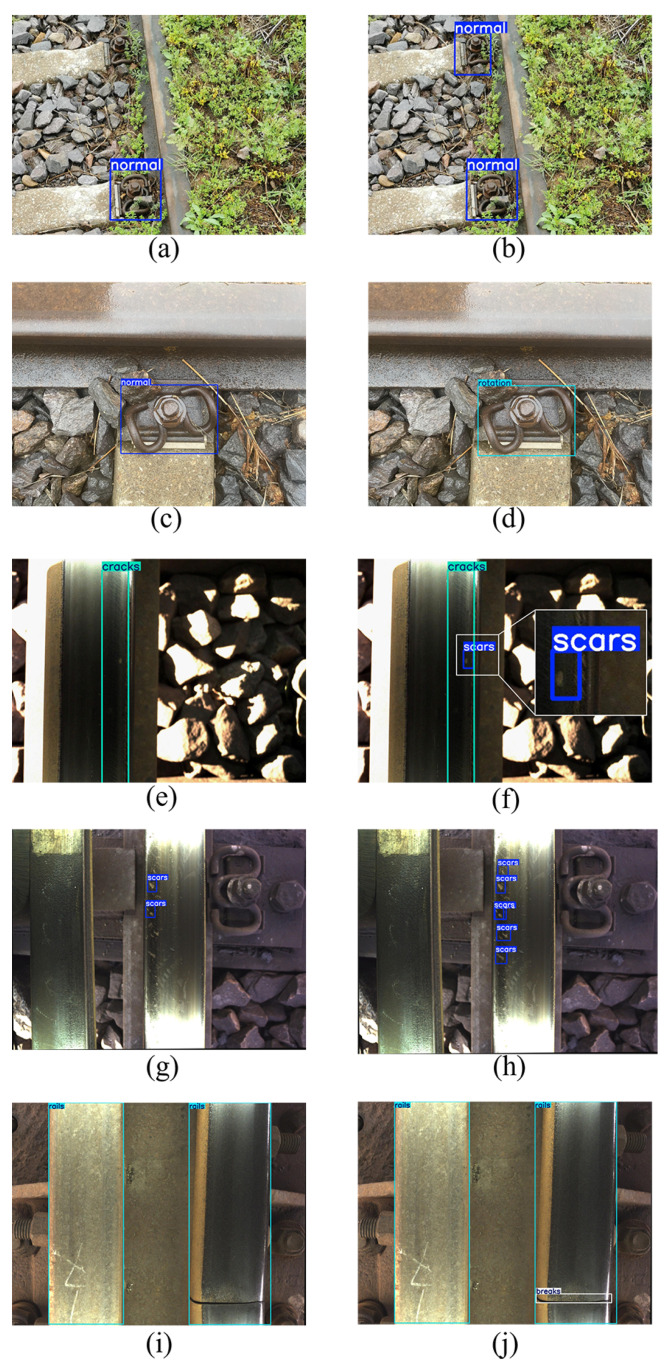
Visual detection results of the original YOLO11 and improved YOLO11. (The subfigures on the left (**a**,**c**,**e**,**g**,**i**) are the results of the original YOLO11 detection, and the subfigures on the right (**b**,**d**,**f**,**h**,**j**) are the results of the improved YOLO11 detection).

The actual detection results of the model are shown in Figure 8. Panels (a), (c), (e), (g), and (i) present the detection results of the original YOLO11 model, while panels (b), (d), (f), (h), and (j) display the results of the improved YOLO11 model. A comparison between Figure 8a,b clearly illustrates that, due to the shooting angle of the visual sensor, the distant fastener appears relatively small in the image. The original YOLO11 model fails to identify this fastener, whereas the improved YOLO11 model accurately recognizes it as a normal fastener. Additionally, in Figure 8c,d, the original YOLO11 incorrectly classifies the rotated fastener as normal, while the improved YOLO11 model successfully categorizes it as a rotated fastener. Similarly, in Figure 8e,j, the improved YOLO11 model demonstrates an enhanced ability to detect small targets. The comparative results indicate that, in contrast to the original YOLO11 model, the improved YOLO11 model significantly reduces false positives and missed detections in track defect detection, and particularly in small target recognition. This improvement leads to a notable increase in detection accuracy, further highlighting the robustness of our model.

Our improved model shows a significant enhancement in recognition capability. Although its storage space increased by approximately 1.1 MB, and the number of parameters increased by about 0.2 million, these improvements are still significant in practical applications, especially in safety-critical fields like railway defect detection. The timely identification and repair of defects can effectively mitigate potential substantial losses. Nevertheless, with the rapid development of AI (Artificial Intelligence) in recent years, the computing power of edge devices is also increasing swiftly, and cloud computing platforms provide scalable resources, which are sufficient to meet the increased computational demands of the model. All the data are shown in Table 6 and Table 7.

#### 3.4.2. Comparison Experiment

To evaluate the effectiveness of the improved model proposed in this paper, we designed a comprehensive comparative experimental scheme. The experiments selected representative algorithms in the field of object detection as baseline models, including the two-stage detector Faster R-CNN [25] and the single-stage detector SSD [26], as well as YOLO v5, YOLOv6, YOLOv8, and the original YOLO11. The experimental data were sourced from three rail inspection datasets with distinct characteristics: the track fastener dataset, the track surface dataset, and the railway track fault detection dataset [27]. In addition to conventional performance metrics such as Precision (P), Recall (R), and Mean Average Precision (mAP), we also introduced frame rate (fps) as a real-time evaluation metric, which was tested on a ordinary laptop equipped with an NVIDIA GeForce RTX 3060 GPU (NVIDIA, Santa Clara, CA, USA). Detailed experimental results are presented in Table 8, Table 9 and Table 10.

In the testing of the track fastener dataset, the experimental data showed that Faster R-CNN achieved an outstanding performance in mean average precision (mAP@0.5), reaching 96.2%, outperforming other algorithms. However, the two-stage detection mechanism of this algorithm significantly increased its computational complexity, resulting in a frame rate of only 12 fps, making it difficult to meet real-time detection requirements in rail scenarios. In contrast, the single-stage SSD algorithm achieved a higher detection frame rate but exhibited noticeable shortcomings in other metrics. Notably, the improved YOLO11 model proposed in this paper demonstrated significant advantages among the YOLO series algorithms, achieving the highest levels in precision (P = 92.2%) and mean average precision (mAP@0.5 = 95.9%, mAP@0.5:0.95 = 74.4%). This advantage was consistently validated in the testing of the other two datasets.

Beyond detection performance, the improved model maintained a compact size and low parameter count. Additionally, the model achieved a detection frame rate of approximately 90 fps, with a single-frame processing time of 11 ms. Each frame captured by our image sensor covers an area of about 0.5 m × 0.5 m. Assuming a 10% overlap area for every 10 images, the model can achieve a rail inspection speed of 40 m per second, equivalent to 145 km per hour. This fully meets the inspection requirements of high-speed railways. These results demonstrate that the optimization strategies proposed in this study effectively achieve an optimal balance between detection accuracy and computational efficiency.

In conclusion, the enhanced method presented in this paper substantially improves the accuracy of the YOLO11 algorithm. Through a series of targeted optimizations, the revised model exhibits exceptional performance across multiple datasets, showcasing stronger generalization capabilities in complex and variable track environments. These findings provide valuable insights and references for future research and lay a solid foundation for further advancements in track safety detection technology.

## 4. Conclusions

This paper presents an algorithm for track defect detection based on the YOLO11 architecture. This enhancement seeks to overcome the shortcomings of conventional track defect detection methods, thereby improving both the efficiency and accuracy of the detection process. By replacing the original convolutions in the YOLO11 backbone with SPD-Conv building blocks, the model’s focus on medium and small targets is significantly enhanced. The introduction of the EffectiveSE attention mechanism, integrated into the backbone network, allows the model to leverage information from features at different layers, thus improving its feature representation capabilities.Furthermore, a small target detection head was incorporated into the neck network to enhance the model’s capacity to identify objects across different scales. This approach also improves the model’s generalization capacity across diverse track scenarios, including track fasteners and track surfaces. In this study, ablation and comparative experiments were conducted using the collected Track Fastener Dataset and Track Surface Dataset. Compared to mainstream models, the enhanced YOLO11 model not only attains superior accuracy but also demonstrates reduced resource consumption, thereby striking a balance between precision and performance, offering an effective solution for the track defect detection industry.

In the future, we will continue to optimize the model by using pruning and quantization techniques to reduce the demand for computational resources while ensuring detection accuracy. For example, we aim to test the effect of pruning different channels on the model and assess the importance of each channel. Additionally, we will employ semantic segmentation networks (such as U-Net) for the pixel-wise classification of track images, allowing for the precise segmentation of defect areas. Through multi-task learning, we will build a shared neural network as a feature-extractor to derive high-level features from the original track images, enabling the model to simultaneously perform defect classification, localization, and segmentation tasks. This approach will further enhance the comprehensiveness and accuracy of detection, promoting the development of the intelligent track inspection field.

## Figures and Tables

**Table 1:** The number of images and instances for each category in the track fastener dataset.

	Normal	Rotation	Lack	Break
images	470	266	171	405
instances	892	268	206	433

**Table 2:** The number of images and instances for each category in the track surface dataset.

	Scar	Rail	Break	Crack	Lightband
images	1485	3428	502	2776	3373
instances	3738	4315	502	3358	4259

**Table 3:** Experimental environment configuration.

Name	Specific Information
Operating System	Ubuntu 20.04 (Canonical, London, UK)
CPU	Intel (R) Xeon(R) Platinum 8163 CPU @ 2.50GHz (Intel, Santa Clara, CA, USA)
GPU	NVIDIA TITAN Xp (12GB) (NVIDIA, Santa Clara, CA, USA)
Pytorch	2.5.0
Python	3.11.10
YOLO	ultralytics-8.3.23 with Docker

**Table 4:** Detection performance of the improved YOLO11 on the track fastener dataset for each category.

	Normal	Rotation	Lack	Break
mAP@0.5/%	96.4	96.2	95.9	95.2

**Table 5:** Detection performance of the improved YOLO11 on the track surface dataset for each category.

	Scar	Rail	Break	Crack	Lightband
mAP@0.5/%	50.4	96.2	99.5	98.5	99.5

**Table 6:** Ablation experiment results of the track fastener dataset.

SPD-Conv	EffectiveSE	Detection Deads	Model Size/MB	Params/M	P/%	R/%	mAP @0.5/%	mAP @0.5:0.95/%
			5.5	2.6	89.8	89.6	94.2	70.3
✓			6	2.8	88.6	86.9	94.5	71.6
	✓		5.7	2.7	92.5	85.7	94.3	71.3
		✓	5.2	2.5	91	89.5	95.2	71.2
✓	✓		6.4	2.8	89.8	90.5	94.5	74
✓	✓	✓	6.6	3	92.2	90.4	95.9	74.4

**Table 7:** Ablation experiment results of the track surface dataset.

SPD-Conv	EffectiveSE	Detection Deads	Model Size/MB	Params/M	P/%	R/%	mAP @0.5/%	mAP @0.5:0.95/%
			5.5	2.6	87.9	88.4	88.7	67.4
✓			6.0	2.8	88.1	89.3	88.9	67.8
	✓		5.7	2.7	88.6	88.6	88.2	67.3
		✓	5.2	2.5	88.0	89.2	88.5	67.5
✓	✓		6.4	2.8	89.2	88.6	88.9	68.0
✓	✓	✓	6.6	3.0	89.1	89.1	89.5	68.6

**Table 8:** Compared with the experimental results of the track fastener dataset.

Algorithms	Model Size/MB	Params/M	P/%	R/%	mAP @0.5/%	mAP @0.5:0.95/%	fps
Faster R-CNN	108.3	28.3	82.7	92.0	96.2	61.9	12
SSD	92.1	24.1	89.4	72.6	88.7	60.4	90
YOLOv5n	5.3	2.5	89.7	90.4	94.1	70.7	122
YOLOv6n	8.7	4.2	90.0	85.8	93.7	72.3	127
YOLOv8n	6.3	3.0	90.7	92.1	95.0	73.1	127
YOLO11n	5.5	2.6	89.8	89.6	94.2	70.3	107
Ours	6.6	3.0	92.2	90.4	95.9	74.4	91

**Table 9:** Comparison with the experimental results of the track surface dataset.

Algorithms	Model Size/MB	Params/M	P/%	R/%	mAP @0.5/%	mAP @0.5:0.95/%	fps
Faster R-CNN	108.3	28.3	75.1	87.9	82.3	56.1	14
SSD	92.6	24.2	84.0	76.5	75.1	50.1	97
YOLOv5n	5.3	2.5	88.6	87.7	88.0	67.1	141
YOLOv6n	8.7	4.2	89.0	87.8	88.5	67.9	166
YOLOv8n	6.3	3.0	88.5	87.2	88.3	67.3	140
YOLO11n	5.5	2.6	87.9	88.4	88.7	67.4	127
Ours	6.6	3.0	89.1	89.1	89.5	68.6	112

**Table 10:** Comparison with the experimental results of the railway track fault dataset.

Algorithms	Model Size/MB	Params/M	P/%	R/%	mAP @0.5/%	mAP @0.5:0.95/%	fps
Faster R-CNN	108.3	28.3	83.6	69.0	79.0	50.1	13
SSD	91.6	24.0	80.2	71.3	79.0	55.2	99
YOLOv5n	5.3	2.5	81.8	70.7	79.6	52.7	124
YOLOv6n	8.7	4.2	82.5	68.3	71.2	42.4	139
YOLOv8n	6.3	3.0	85.4	66.2	78.7	49.9	139
YOLO11n	5.5	2.6	84.8	68.6	81.3	55.8	98
Ours	6.6	3.0	85.9	71.8	81.3	58.2	89

## Data Availability

The datasets presented in this article are not readily available because confidentiality agreements with project partners.

## Abbreviations

The following abbreviations are used in this manuscript:

YOLO	You Only Look Once
SPD-Conv	Spatial Pyramid Dilation Convolution
EffectiveSE	Effective Squeeze-Excitation
CNN	Convolutional Neural Network
SPFF	Spatial Pyramid Pooling Fast
PAN	Path Aggregation Network
TP	True Positive
FP	False Positive
AP	Average Precision
AI	Artificial Intelligence
